# Insights from the molecular docking aided interaction analysis of HfQ with small RNAs

**DOI:** 10.6026/97320630018425

**Published:** 2022-04-30

**Authors:** Rajkumar Praveen, Johni Rexliene, Ashwini Karuppaswamy, Murugesan Rajeshkannan, Viswanathan Balaji, Jayavel Sridhar

**Affiliations:** 1 Department of Biotechnology (DDE), Madurai Kamaraj University, Madurai-625021, Tamilnadu, India

**Keywords:** HfQ, small RNA, Co-IP, RNA-SEQ, reads

## Abstract

Hfq, RNA binding protein, is widely found in most of the prokaryotes. It plays a key role in gene regulation by binding with small RNA and facilitates mRNA pairing there by suppress or boost translation according to RNA structures. Interaction between sRNAs and HfQ in Salmonella SL1344 were screened using Co-Immuno Precipitation (HfQ-CoIP) studies earlier. We have formulated an In silico approach, to model the 3D structures of 155 sRNA and studied their interactions with HfQ proteins. We have reported the key interacting PHE42, LEU7, VAL27, PHE39 and PRO21 residues of HfQ binds with many small RNAs. Further mutation of PHE42 in to ALA42 in HfQ leads to loss of sRNA binding efficiency. We have differentiated the interactions in to HfQ binding and non-binding sRNAs, based on Atomic Contact Energy and area. This methodology may be applied generically for functional grouping of small RNAs in any organism.

## Background:

HfQ is a homo hexameric protein widely found in many Bacteria and Archaea [1]. HfQ was initially identified as host factor in replication process of Qβ RNA bacteriophage [2]. Later, it was found to involve in the virulence of Salmonella typhimurium [3]. Hfq could be identified as post transcriptional regulator for small RNA-mRNA complexes in many Bacteria [4]. Hfq and HfQ dependent small RNAs and their network are major contributors of virulence and development in Salmonella [5]. Hfq suppress the translation by guarding sRNA so that it binds to 5' region of mRNA, thereby making 5' region inaccessible [4]. HfQ and RNAseE binds with similar region on RNA which facilitates coupled degradation of sRNA and its target mRNA in Escherichia coli [6]. Hfq promotes protein synthesis in similarly by directing sRNA to bind to 5' region of mRNA, in cases when 5' region of its mRNA forms a secondary structure which inhibit ribosome binding [7]. In such cases, secondary structure of mRNA is disrupted by the action of Hfq and sRNA. Hfq protects sRNA from ribonuclease cleavage while it can also promote mRNA cleavage [8]. Hfq's role seems to depend upon the structural information encoded in the RNA molecules which interacts with Hfq [4],[6]. Hfq (PDB ID: 2YLB) has ring like architecture and it has two faces for potential interaction with nucleic acids such as proximal face and distal face (Figure 1 - see PDF). The polymer chains of 2 YLB from N-terminus and C-terminal were marked from blue to red color (Figure 2 - see PDF). Face on which alpha helix exposed and near to centre is called proximal face and the one opposite to it is called distal face [9]. sRNAs interact with proximal and distal face differently. Hfq have highly conserved motif among 26 bacterial genomes. VYKHAIST are reported as the conserved motif in the Hfq in diverse genera [10]. Many Co-Immunno Precipitation studies show HfQ interacting with sRNAs. Therefore, it is of interest to document derived data from the molecular docking aided interaction of HfQ with small RNAs to gain functional insights.

## Materials & Methods:

Already reported 155 sRNA sequences from SL1344 through Co-IP studies [3] were taken. Corresponding sequences were obtained and then 3D structures of sRNA were predicted using RNAComposer (https://rnacomposer.cs.put.poznan.pl/) [11]. With help of Literature survey, sRNAs which were supposed to bind with Hfq were identified and similarly non interacting sRNAs were also listed.

## Protein-RNA docking:

PatchDock server was used to carry out the Protein-RNA docking. PatchDock returns Z score and Atomic contact Energy (ACE) for the complex [12], [13]. PatchDock server is based upon geometry-based molecular docking algorithm [13]. PatchDock provides docking transformations that yield good molecular shape complementarities. Transformations had wide interface areas and small amounts of steric clashes. PatchDock algorithm divides the Connolly dot surface, a representation of surface where only atoms that are accessible to solvent [14] are shown, into concave, convex and flat patches.

Protein RNA interface of complex is identified using PRIDB of IOWA State University [15]. Conformations are generated by matching complementary patches. Scoring function evaluates each candidate transformation that considers both geometric fit and atomic desolvation. Based on geometric shape complementary score and conformations are ranked. Atomic contact energy is the desolvation free energy required to transfer atom from water to a protein's interior. Approximate interface area of the Protein-RNA complex is returned as Area. sRNA passing through the pore of Hfq were examined using VMD [16] and the results were tabulated.

## Results & Discussion:

Among the interacting residues, Phe42 of Hfq interacts well with sRNA. Residues interacting with sRNA are Phenyl alanine, Leucine, Glycine, Isoleucine, Valine, Proline, Alanine, Methionine which are hyphobic and non-polar in nature. In most of the interactions, Phe42 is interacting sRNA. Phe42 forms a loop in Hfq protein. Hfq highly binds with AU rich regions of sRNA. Most of the interacting residues of sRNA of Hfq were PHE42, LEU7, VAL27, PHE39, and PRO21. This study has found Phe42 as a key residue for sRNA interactions. Alteration of Phe42 position with Ala42 has decreased the binding of Hfq with small RNAs and rpoS mRNA segment containing translation initiation region. There by marking the suppression of the phenotype [17]. On the other way, sRNA having cDNA reads Hfq coIP having lesser score. When the interacting residues were analyzed on hydrophobic interaction, Phenyl alanine takes place in most of the interaction with length less than 5 Å resolution. As suggested earlier Phe42 and Phe39 were also important in virulence of Salmonella. Tyr42 plays a key role in RNA binding Hfq by stacking with the RNA bases in Staphylococcus aureus [17]. Entire Salmonella, genera has Phe at 42nd position. Both Tyr and Phe amino acids are Aromatic amino acids and similar in structure wise.

PatchDock Score and Deep sequencing reads were compared and sixteen sRNAs were proposed to interact with HfQ from this study (Table 1 - see PDF). The data are taken and analyzed with positive control shown with earlier Co-IP studies [18] (Table 2 - see PDF). IsrC sRNA exhibits less Hfq coIP reads but not having fewer score in PatchDock score (Table 3 - see PDF). But it is not the case with InvR, where it holds highest Patch Dock score among the Binding group sRNA and it also have high Hfq coIP read. Taking Patch dock score of InvR, sRNA of unknown group, with high scores which were highlighted in table are more likely to have interaction with Hfq. Those high scoring sRNA candidates are NC_016810_1_4553813_4553972, NC_016810_1_1466054_1466240, NC_016810_1_1784673_1784725, gi_378697983_c3312520_3312429, Stnc2090, Stnc1270, gi_378697983_c1913755_1913615, gi_378697983_c756436_756377 (Table 4 - see PDF). Other sRNA might have activity with Hfq, but sRNA not binding also has relatively good patchdock score comparing with other sRNA in binding group. But our interrogation studies has found too many false negatives among non-binding sRNA group. It is evident from certain sRNA has high PatchDock score also have high interface area of Protein-RNA complex. Hence only sRNA with very high PatchDock scores is proposed to have interaction with Hfq. The Hfq-RNA structure has binding mode of the RNA within the central basic pore of Hfq. Binding of HfQ through central pore or along the central groove having electrostatic interaction, Hfq modulating RNA structure, thereby regulating translation, can be suggested [17]. This study has attempted to identify the key interacting residues of HfQ having binding with sRNAs. We have differentiated the HfQ binding and non-binding sRNAs based on their Atomic Contact Energy and Area value thresholds.
